# Identification of New Regulators of Pancreatic Cancer Cell Sensitivity to Oxaliplatin and Cisplatin

**DOI:** 10.3390/molecules27041289

**Published:** 2022-02-14

**Authors:** Vera Skripova, Ramilia Vlasenkova, Yan Zhou, Igor Astsaturov, Ramziya Kiyamova

**Affiliations:** 1 “Biomarker” Research Laboratory, Institute of Fundamental Biology and Medicine, Kazan Federal University, 420008 Kazan, Russia; vsskripova@gmail.com (V.S.); r.mukhamadeeva@yandex.ru (R.V.); 2 Institute for Cancer Research, Fox Chase Cancer Center, Philadelphia, PA 19111, USA; yan.zhou@fccc.edu (Y.Z.); igor.astsaturov@fccc.edu (I.A.)

**Keywords:** pancreatic cancer, chemotherapy resistance, oxaliplatin, cisplatin, CRISPR/Cas9 screening, biomarkers

## Abstract

The chemoresistance of tumor cells is one of the most urgent challenges in modern oncology and in pancreatic cancer, in which this problem is the most prominent. Therefore, the identification of new chemosensitizing co-targets may be a path toward increasing chemotherapy efficacy. In this work, we performed high-performance in vitro knockout CRISPR/Cas9 screening to find potential regulators of the sensitivity of pancreatic cancer. For this purpose, MIA PaCa-2 cells transduced with two sgRNA libraries (“cell cycle/nuclear proteins genes” and “genome-wide”) were screened by oxaliplatin and cisplatin. In total, 173 candidate genes were identified as potential regulators of pancreatic cancer cell sensitivity to oxaliplatin and/or cisplatin; among these, 25 genes have previously been reported, while 148 genes were identified for the first time as potential platinum drug sensitivity regulators. We found seven candidate genes involved in pancreatic cancer cell sensitivity to both cisplatin and oxaliplatin. Gene ontology enrichment analysis reveals the enrichment of single-stranded DNA binding, damaged DNA binding pathways, and four associated with NADH dehydrogenase activity. Further investigation and validation of the obtained results by in vitro, in vivo, and bioinformatics approaches, as well as literature analysis, will help to identify novel pancreatic cancer platinum sensitivity regulators.

## 1. Introduction

Pancreatic ductal adenocarcinoma (PDAC) is one of the most aggressive types of cancer. Despite the low incidence of the disease, PDAC ranks second or third among the causes of death from oncological diseases [1] and it has one of the lowest five-year survival rates, being less than 10% [1,2]. The main reason is that the asymptomatic early stages of the disease result in a late diagnosis at unresectable stages [3,4]. Another cause of the severity of PDAC is the high rate of tumor resistance to chemotherapeutic drugs limiting the drug treatment options [5]. Unfortunately, new promising approaches to the treatment of oncological diseases, including immunotherapy, have not shown effectiveness in the treatment of PDAC [1,6]. Currently, combined chemotherapy regimens are used to treat metastatic PDAC, including FOLFIRINOX (leucovorin, 5-FU, irinotecan, and oxaliplatin) and the combination of gemcitabine with other cytotoxic agents, such as platinum-based oxaliplatin and cisplatin [6,7]. PARP inhibitors, including their combinations with platinum drugs, were actively studied as part of the clinical trials for PDAC patients with mutations in the BRCA1/2 genes [8,9], which occur in 5–9% of PDAC cases [10,11].

The low effectiveness of the chemotherapy regimens used in the treatment of PDAC is due to the complexity of the disease, including the genetic heterogeneity of tumors, the structure, and the organization of the tumor microenvironment [12,13,14]. Currently, more attention is given to an individual approach to select a PDAC treatment strategy, considering the molecular features of the tumor, including mutations in significant genes [6,15]. For example, platinum-based drugs can be used to treat PDAC patients with mutations in genes associated with DNA repair, but there are not enough data to assess their effectiveness [6,16]. At the moment, the study of the possible mechanisms of regulation of PDAC sensitivity to platinum drugs is limited, and it is discussed mainly in the context of well-known markers of DNA repair pathway defects, such as mutations in the BRCA1/2 and PALB2 genes [15,16,17].

Thus, the search for new regulators of the sensitivity of PDAC to platinum drugs (predictive biomarkers) is extremely relevant for expanding the options of PDAC drug therapy.

High-performance technologies, including SEREX (Serological Analysis of Recombinant Tumor cDNA Expression Libraries), SERPA (Serological Proteome Analysis), and CRISPR/Cas9 technologies [18,19,20,21], make it possible to search for biomarkers among numerous candidates in a relatively short time. CRISPR/Cas9 genome editing technology, applied in the format of high-performance screening using guide RNA libraries, allows analysis of the effect of multiple genes on cell viability in various conditions, including drug treatment [22,23,24,25,26].

In our work, we performed high-performance synthetic lethal knockout CRISPR/Cas9 screenings to search for new regulators of sensitivity of the pancreatic cancer cells MIA PaCa-2 to oxaliplatin and cisplatin using two sgRNA libraries, one of which targets 4799 cell cycle and nuclear protein genes, while the other genome-wide library targets 18,166 genes. This approach allowed us to simultaneously analyze the influence of thousands of genes on the cell sensitivity to platinum drugs and identify new possible regulators of chemosensitivity and new therapeutic approaches to increase the effectiveness of the studied drugs.

## 2. Results

The synthetic lethal knockout CRISPR/Cas9 screenings of human pancreatic cancer MIA PaCa-2 cells were performed to identify genes potentially involved in the regulation of the sensitivity to cisplatin and oxaliplatin. MIA PaCa-2 cells expressing doxycycline-inducible Cas9 [27] were separately transduced with two sgRNA lentivirus libraries targeting 4799 genes of cell cycle and nuclear protein genes (“CCN”), and genome-wide targeting 18,166 genes (“GW”) (Table 1, Tables S1 and S2).

**Figure 1:**
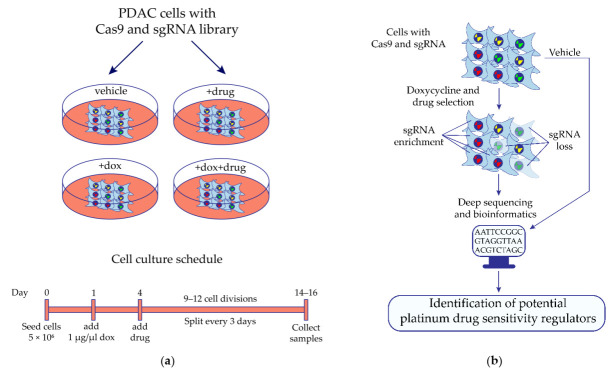
Identification of potential regulators of MIA PaCa-2 pancreatic cancer cell sensitivity to platinum drugs by the synthetic lethal knockout CRISPR/Cas9 screening. (**a**) The design of the synthetic lethal knockout CRISPR/Cas9 screening of pancreatic ductal adenocarcinoma (PDAC) cell line MIA PaCa-2. (**b**) Application of the high-throughput synthetic lethal knockout CRISPR/Cas9 screening to identify potential predictive biomarkers. Cells expressing doxycycline-inducible Cas9 and transduced with the lentiviral sgRNA library are cultivated in the presence or absence of the studied drug. If Cas9-mediated knockout of the gene leads cells to death in the presence of the drug, then representation of the corresponding sgRNA has decreased in the cell population, and vice versa compared with the control conditions. Differently presented sgRNA are identified by deep sequencing of the sgRNA-containing region of cell genomic DNA integrated during lentiviral transduction of cells with the sgRNA library. Bioinformatics and statistical approaches are used to find gene knockout which significantly changed the sensitivity of cells to the studied drug. Dox—doxycycline.

Cisplatin was used to treat cells infected with the cell cycle/nuclear proteins sgRNA lentiviral library, which includes DNA damage response (DDR) genes associated with the main mechanism of cisplatin cytotoxicity. Treatment with oxaliplatin was applied to the “CCN” as well as the “GW” sgRNA library expressing cells due to the evidence that oxaliplatin possibly has mechanism of action unrelated to DDR [28,29,30]. Thus, three independent CRISPR/Cas9 screenings were performed during this work. Cells were cultivated with a sublethal concentration of drugs or under control conditions for 9–12 cell divisions and collected for further analysis (Figure 1a). Representation of sgRNAs in the cell population changes depending on treatment conditions: it decreases if knockout of the corresponding gene leads to cell death, and vice versa compared with the control conditions. Deep sequencing can be used to identify differently presented sgRNAs due to the sgRNA-containing region being integrated in cell genomic DNA during lentiviral transduction with the sgRNA library (Figure 1b) [31]. The abundance of sgRNA in cells cultivated under different conditions was analyzed according to Wang and Parnas [26,32].

To identify genes whose knockout leads to MIA PaCa-2 cell platinum sensitivity changes, the abundances of sgRNAs in samples treated with 1 ug/ul of doxycycline (“dox”) were compared with those of samples treated with 1 ug/ul doxycycline in combination with 1 ug/ul of oxaliplatin or 3 ug/ul of cisplatin (“dox + drug”). The sgRNAs for which abundance was significantly (FDR (False Discovery Rate) ≤ 0.05) changed in two and more folds were considered as “effective”. The “effective” sgRNAs revealed in the “vehicle vs. drug” comparison of non-treated samples (vehicle, “veh”) or treated with the drug only (“drug”) were considered as false positive and excluded from further analysis. A gene was considered a candidate if abundances of two or more corresponding “effective” sgRNAs changed in the same direction. Candidates from “veh vs. dox” comparisons were considered as essential genes and were excluded from “dox vs. dox + drug” comparison results if present. Candidate genes were analyzed by The Comparative Toxicogenomics Database (CTD) and a database of genes related to platinum resistance in cancer [33] to identify genes previously associated with cancer platinum drug resistance.

The results of the individual CRISPR/Cas9 screenings performed are presented in Table 2, Tables S3–S5. As a result of oxaliplatin CRISPR/Cas9 screenings with the “CCN” sgRNA library, we identified eight candidate genes, the knockout of which significantly changed the sensitivity of MIA PaCa-2 cells to the oxaliplatin (*BRIP1*, *ERCC4*, *FANCD2*, *FANCG*, *FANCI*, *MAD2L2*, *PPP2R2A*, *NPEPPS)* (Table 2). Of these, seven genes (*BRIP1*, *ERCC4*, *FANCD2*, *FANCG*, *FANCI*, *MAD2L2*, *PPP2R2A*) were previously related to the platinum sensitivity/resistance of different types of cancer cells, with *NPEPPS* being reported for the first time as a platinum drug sensitivity regulator.

CRISPR/Cas9 screenings with oxaliplatin and the genome-wide sgRNA library revealed 141 candidate genes whose knockout influenced MIA PaCa-2 cell sensitivity to oxaliplatin (Table 2). Among them, 13 genes (*ALDH1A1*, *CDKN1B*, *FANCD2*, *GAS7*, *GCLM*, *LRP1*, *MAD2L2*, *MAPK14*, *MCM9*, *NDUFS8*, *STK11*, *TNFRSF12A*, *UBE2T*) were previously associated with platinum resistance/sensitivity and 128 genes are reported as platinum sensitivity regulators for the first time.

The results of the CRISPR/Cas9 screening of MIA PaCa-2 cells with cisplatin and the sgRNA library targeting cell cycle and nuclear proteins show that the knockout of 34 genes significantly changed the drug sensitivity of PDAC cells (Table 2). Thirteen genes (*BRCA1*, *BRIP1*, *ERCC4*, *EXO1*, *FANCD2*, *FANCG*, *FANCI*, *MAD2L2*, *NBN*, *PMS2*, *RAD51B*, *TXNRD1*, *XRCC2*) were previously associated with platinum sensitivity/resistance and 21 genes are shown as regulating platinum sensitivity of cancer cells for the first time.

So, three CRISPR/Cas9 screenings conducted in this work allow us to identify 173 candidate genes potentially involved in the regulation of MIA PaCa-2 cell sensitivity to the platinum drugs oxaliplatin and/or cisplatin (Table 3). Of the 173 candidate genes, 25 were previously related to platinum sensitivity/resistance according to CTD and Huang [33] and 148 genes are reported for the first time as platinum sensitivity regulators. We found seven genes involved in the regulation of the MIA PaCa-2 cell sensitivity to both oxaliplatin and cisplatin: *BRIP1*, *ERCC4*, *FANCD2*, *FANCG*, *FANCI*, *MAD2L2*, *NPEPPS*. Among them, the *NPEPPS* has not yet been reported as associated with platinum sensitivity regulation in the literature. Notably, *FANCD2* and *MAD2L2* were revealed as platinum sensitivity regulators in all three CRISPR/Cas9 screenings performed.

**Figure 2:**
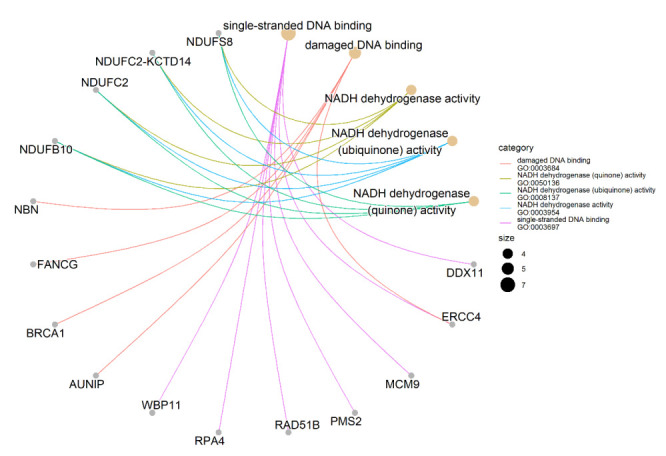
Gene-concept network of identified genes. It shows the enriched concepts, obtained with a *p*-value cut-off of 0.05 (Gene Ontology biological processes) linked to the genes involved in each concept, and the relationship between them when genes are involved in more than one process. The size of the concept nodes depends on the gene count involved in that pathway.

We performed the Gene Ontology (GO) enrichment analysis of 173 identified candidate genes to characterize their possible biological functions. Only 152 out of 173 candidate genes are annotated in the GO database. We found the significant enrichment of single-stranded DNA binding in seven genes (GO:0003697: *DDX11*, *ERCC4*, *MCM9*, *PMS2*, *RAD51B*, *RPA4*, *WBP11*); damaged DNA binding in five genes (GO:0003684: *AUNIP*, *BRCA1*, *ERCC4*, *FANCG*, *NBN*); and NADH dehydrogenase activity in four genes (GO:0003954, 0008137, 0050136, 0003955: *NDUFB10*, *NDUFC2*, *NDUFC2-KCTD14*, *NDUFS8*) (Figure 2; Table S6). Among them, *DDX11*, *RPA4* and *WBP11* (single-stranded DNA binding), *AUNIP* (damaged DNA binding), and *NDUFB10*, *NDUFC2*, and *NDUFC2-KCTD14* (NADH dehydrogenase activity) are shown as potential platinum sensitivity regulators for the first time.

Taken together, we identified 173 candidate genes as potential regulators of the PDAC cell sensitivity to platinum drugs (Table 3). Among them, 148 genes were related to platinum drug sensitivity for the first time and 25 genes have already been reported as involved in platinum sensitivity/resistance. Seven candidate genes were involved in the MIA PaCa-2 PDAC cell sensitivity to both cisplatin and oxaliplatin. GO enrichment analysis reveals the single-stranded DNA binding and damaged DNA binding ontologies enriched with five and seven genes, respectively, as well as four ontologies associated with NADH dehydrogenase activity, each with the same four genes (Figure 2, Table S6).

## 3. Discussion

The resistance of tumor cells to chemotherapeutic drugs is one of the most urgent challenges in modern oncology and in pancreatic cancer, for which this problem is the most prominent [14,34,35]. Therefore, the identification of chemosensitizing co-targets may be a path toward increasing chemotherapy efficacy.

In this work, we applied CRISPR/Cas9 genome editing technology as a high-throughput screening to search for genes involved in the regulation of the sensitivity of the pancreatic cancer cell line MIA PaCa-2 to the platinum drugs oxaliplatin and cisplatin. These drugs are used in clinical practice for the treatment of metastatic pancreatic cancer in the combination chemotherapy regimen FOLFIRINOX and in combination with gemcitabine [6,7].

We identified 173 candidate genes whose knockout altered the sensitivity of pancreatic cancer cells MIA PaCa-2 to oxaliplatin and/or cisplatin (Table 3). Out of these, 148 genes are reported as platinum drug sensitivity regulators for the first time and 25 genes have previously been associated with the sensitivity/resistance of tumor cells to platinum drugs, according to the CTD database and published data [33]. Among them, there are genes with products involved in DNA repair (*BRCA1*, *BRIP1*, *ERCC4*, *EXO1*, *FANCD2*, *FANCG*, *FANCI*, *MCM9*, *NBN*, *PMS2*, *RAD51B*, *STK11*, *UBE2T. XRCC2*), cell cycle regulation (*CDKN1B*, *GAS7*, *MAD2L2*, *PPP2R2A*), components of detoxification and antioxidant systems (*ALDH1A1*, *GCLM*, *TXNRD1*), as well as other regulators of the most important intracellular signaling pathways (*LRP1*, *MAPK14*, *NDUFS8*, *TNFRSF12A*).

Interestingly, the knockout of seven genes (*BRIP1*, *ERCC4*, *FANCD2*, *FANCG*, *FANCI*, *MAD2L2*, *NPEPPS)* increased the sensitivity of MIA PaCa-2 cells to both cisplatin and oxaliplatin, including *FANCD2* and *MAD2L2* being revealed as candidates in all three screenings. The products of six out of seven genes are involved in DNA repair and cell cycle regulation processes and the regulation of sensitivity/resistance of different types of cancer cells to platinum drugs, which implies their perspectives for pancreatic cancer. The *NPEPPS* gene is reported as a platinum sensitivity regulator in this work for the first time. This gene encodes puromycin-sensitive aminopeptidase, suggested to be involved in cell cycle processes [36].

The results of the GO enrichment analysis showed the enrichment of pathways associated with DNA repair, single-stranded DNA binding (GO: 0003697), damaged DNA binding (GO: 0003684), and NADH dehydrogenase activity (GO: 0003954, 0008137, 0050136, 0003955) (Figure 2). The enrichment of the first two pathways is predictable, considering known mechanisms of platinum cytotoxicity [37,38]. There are both previously known (*ERCC4*, *MCM9*, *PMS2*, *RAD51B*, *BRCA1*, *ERCC4*, *FANCG*, *NBN*), and first identified in this work (*DDX11*, *RPA4*, *WBP11*, *AUNIP*) regulators of sensitivity to platinum drugs.

The NADH dehydrogenase complex is a key component of cellular respiration and oxidative phosphorylation, as well as a regulator of the NAD^+^/NADH ratio. It is known that impaired function of the NADH dehydrogenase complex and a decreased level of NAD^+^ lead cells to acquire more aggressive phenotypes and a high metastatic potential [39]. In addition, a decreased content of NAD^+^ was observed in tumor cells resistant to cisplatin [40,41]. In this work, we observed that knockout of the NADH dehydrogenase complex subunit genes *NDUFB10*, *NDUFC2*, *NDUFC2-KCTD14*, *NDUFS8* led to a decrease in the sensitivity of MIA PaCa-2 pancreatic cancer cells to oxaliplatin. It should be noted that *NDUFB10*, *NDUFC2*, and *NDUFC2-KCTD14* are first identified by us as potential regulators of sensibility to platinum drugs, while *NDUFS8* was previously associated with sensitivity/resistance to cisplatin, according to the CTD database. Consequently, the NADH dehydrogenase complex can be considered as a potential target for the development of approaches to increasing the effectiveness of platinum drugs and one of the components of a possible platinum resistance mechanism.

The fact that our candidate gene list includes those previously associated with sensitivity/resistance to platinum drugs, as well as being involved in platinum cytotoxicity, indicates the adequacy of the CRISPR/Cas9 screening approach to search for regulators of drug sensitivity.

Thus, the approach we used made it possible to identify 173 candidate genes whose knockout altered the sensitivity of MIA PaCa-2 pancreatic cancer cells to cisplatin and/or oxaliplatin, including 25 genes previously reported as involved in the platinum drugs sensitivity/resistance of different type of cancer cells. According to our results, the knockout of seven genes led to the increase in the MIA PaCa-2 PDAC cell sensitivity to both oxaliplatin and oxaliplatin. In this work, 148 genes were characterized as regulators of the sensitivity of tumor cells to platinum drugs. Among them, genes involved in the processes of single-stranded DNA binding (*DDX11*, *RPA4* and *WBP11*), damaged DNA binding (*AUNIP*), NADH dehydrogenase activity (*NDUFB10*, *NDUFC2*, *NDUFC2-KCTD14*), and cell cycle processes, such as *NPEPPS,* are of the most interest and can be considered as potential targets for the development of approaches to increase the effectiveness of pancreatic cancer treatment with platinum drugs. The results obtained in this work need to be further investigated and validated by in vitro, in vivo and bioinformatics approaches, as well as literature searching for the identification of novel pancreatic cancer platinum sensitivity regulators.

## 4. Materials and Methods

### 4.1. Cell Culture and Reagents

The pancreatic adenocarcinoma cell line MIA PaCa2 expressing doxycycline-inducible Cas9 (MIA PaCa2/Cas9) obtained in our previous work [27] was grown in RPMI-1690 media supplemented with 10% fetal bovine serum, L-glutamine, penicillin, streptomycin and 1 ug/mL puromycin. The cells were transduced with lentiviral sgRNA libraries according to Wang et al., 2014 [26]. The number of cells handled was 5 × 10^6^ to provide 100× and 55× coverage of the “Cell cycle/nuclear proteins” and “Genome-wide” sgRNA libraries, respectively. The doxycycline hyclate, oxaliplatin and cisplatin were purchased from Merck (Darmstadt, Germany).

### 4.2. sgRNA Libraries

We used two sgRNA libraries: (1) ”Cell cycle/nuclear proteins genes” targeting 4799 genes of cell cycle and nuclear proteins (“CCN”, 50,000 sgRNAs) and (2) ”Genome-wide” targeting 18,166 genes (“GW”, 90,000 sgRNAs). The “CCN” sgRNA library was taken from Addgene and the “GW” sgRNA library was synthesized by CustomArray Inc (Redmond, WA, USA). The sequences of sgRNAs were taken from Wang et al., 2014 [26].

### 4.3. In Vitro CRISPR/Cas9 Screening

In vitro CRISPR/Cas9 screenings were performed as described previously [26]. Briefly, MIA PaCa2/Cas9 cells transduced with the appropriate lentiviral sgRNA library were cultured with 1 ug/mL doxycycline (dox) for 72 h to induce Cas9 expression prior to the drug treatment. Cells were then cultivated in the presence or absence of oxaliplatin (1 uM) or cisplatin (3 uM) for 9 cell divisions (12 days). The experimental conditions were “veh” (no dox, no drug); “dox” (1 ug/mL dox, no drug); “drug” (no dox, 1 uM oxaliplatin or 3 uM cisplatin); and “dox + drug” (1 ug/mL dox, 1 uM oxaliplatin or 3 uM cisplatin). On the last day of the experiment, cells were collected and genomic DNA was extracted using a Qiagen Gentra Puregene Kit (Hilden, Germany). sgRNA-containing regions of genomic DNA were amplified and barcoded by PCR. Deep sequencing was performed to analyze the sgRNA abundance in cells cultivated under the different conditions. Sequencing data were treated according to Parnas et al., 2015 [32].

### 4.4. Gene Ontology Enrichment Analysis

Gene Ontology analysis was conducted using the clusterProfiler package [42] for exploring and determining the possible biological functions of gene candidates. The significance level was *p* < 0.05. We employed R software to draw the gene-concept network plot.

## Figures and Tables

**Table 1:** Characteristics of the sgRNA libraries used for CRISPR/Cas9 screenings of MIA PaCa-2 pancreatic cancer cells.

Name of sgRNA Library	Number of sgRNA ^1^	sgRNA Per Gene	Number of Target Genes
Cell cycle/ nuclear proteins genes (“CCN”)	50,000	up to 52	4799
Genome-Wide (“GW”)	90,000	4–5	18,166

^1^ Sequences of sgRNAs are presented in the Supplementary Materials Table S1 for “CCN” and Table S2 for “GW” sgRNA libraries (Supplementary Materials).

**Table 2:** Candidate genes changing sensitivity of MIA PaCa-2 pancreatic cancer cells to cisplatin or oxaliplatin according to results of the individual CRISPR/Cas9 screenings with cell cycle/nuclear proteins and genome-wide sgRNA libraries (showing genes for which at least 2 sgRNAs changed representation in ≥ 2 folds, FDR (false discovery rate) ≤ 0.05).

Candidate genes changing sensitivity of MIA PaCa-2 pancreatic cancer cells to oxaliplatin, *n* = 146	
	**CRISPR/Cas9 screening with cell cycle/nuclear proteins sgRNA library**
	knockout led to increase in sensitivity to oxaliplatin (*n* = 7):
	*BRIP1* *, ERCC4, **FANCD2*** *, FANCG, FANCI, **MAD2L2*** *, **NPEPPS***
	knockout led to decrease in sensitivity to oxaliplatin (*n* = 1):
	*PPP2R2A*
	**CRISPR/Cas9 screening with genome-wide sgRNA library**
	knockout led to increase in sensitivity to oxaliplatin (*n* = 96):
	*ACOX1*, *AP2M1*, *ATF6B*, *ATF7IP2*, *BPI*, *BTK*, *C14orf93*, *C17orf70*, *CDC42EP3*, *CDKN1B*, *CERS3*, *CERS6*, *COX7A2L*, *CRYBB1*, *DDAH1*, *DDX27*, *DEFA6*, *EMC2*, *EPB41L3*, *ESCO1*, *FAM160A2*, *FAM209A*, ***FANCD2***, *FCHO2*, *FGFRL1*, *FOCAD*, *GCLM*, *GFOD2*, *GJB6*, *GNAQ*, *GNGT1*, *H2BFWT*, *HERC6*, *HEYL*, *HIST1H1T*, *HMG20B*, *HMGCS2*, *HSD3B2*, *KDM2B*, *KRTAP10-8*, *LEFTY1*, *LGI2*, *LMAN1*, *LPO*, *LRP1*, *LRRC26*, ***MAD2L2***, *MAN2B1*, *MAP7D1*, *MAPK15*, *MARCKSL1*, *MCM9*, *MDN1*, *MEF2A*, *METRN*, *MPLKIP*, *MYO1G*, *NGLY1*, *NIPSNAP3B*, ***NPEPPS***, *NPRL2*, *ORC6*, *PAX7*, *PCGF1*, *PDIA2*, *PEF1*, *PIEZO1*, *PKD2L2*, *PLAGL2*, *PPY*, *PRF1*, *PRRC2A*, *PTBP2*, *PVRL4*, *RASSF5*, *RGL2*, *RPA4*, *RSPH10B*, *SEMA3G*, *SMC5*, *SMOX*, *SMR3B*, *SYNGR2*, *TEKT2*, *TMEM185B*, *TPM3*, *TRIM4*, *UBE2N*, *UBE2T*, *UBE2V2*, *UNC5B*, *VCY*, *VCY1B*, *WBP11*, *ZNF474*, *ZNF804A*
	knockout led to decrease in sensitivity to oxaliplatin (*n* = 45):
	*AGO1*, *AGO2*, *ALDH1A1*, *ANKHD1*, *ATP5O*, *C14orf105*, *CAMTA2*, *CCDC102B*, *CLDN8*, *CREBL2*, *DDX26B*, *DYRK1A*, *EYA3*, *GAS7*, *GATS*, *GDAP1L1*, *GPX8*, *LCA5L*, *LGSN*, *LOC100127983*, *MAPK14*, *MED29*, *MIS18A*, *MRPL51*, *MRPS26*, *MYOZ2*, *NDUFB10*, *NDUFC2*, *NDUFC2-KCTD14*, *NDUFS8*, *NUDCD2*, *PPAPDC2*, *RBBP7*, *RECK*, *RIPPLY3*, *SLC38A2*, *SMAD2*, *STK11*, *TEAD2*, *TLL1*, *TMC5*, *TNFRSF12A*, *ZBTB18*, *ZNF333*, *ZNF536*
**Candidate genes changing sensitivity of MIA PaCa-2 pancreatic cancer cells to cisplatin, *n* = 34**	
	**CRISPR/Cas9 screening with cell cycle/nuclear proteins sgRNA library**
	knockout led to increase in sensitivity to cisplatin (*n* = 32):
	*AUNIP*, *BRCA1*, *BRIP1*, *CAB39*, *CDCA5*, *DBF4*, *DDX11*, *ERCC4*, *ESCO2*, *EXO1*, *FANCD2*, *FANCG*, *FANCI*, *FBXW7*, *INCA1*, *KPNA2*, *MAD2L2*, *MND1*, *NBN*, *NCAPG2*, *NEUROD6*, *NPEPPS*, *PLEKHA7*, *PPP1R12A*, *PSMC3IP*, *PSME3*, *RAD51B*, *RAD9A*, *RHNO1*, *STRA13*, *XRCC2*, *ZNF318*
	knockout led to decrease in sensitivity to cisplatin (*n* = 2):
	*PMS2*, *TXNRD1*

Underlined genes were previously related to platinum drug sensitivity/resistance. Genes highlighted in bold were considered as candidates in both oxaliplatin CRISPR/Cas9 screenings.

**Table 3:** Summary results of the performed CRISPR/Cas9 screenings of MIA PaCa-2 pancreatic cancer cells with cell cycle/nuclear proteins and genome-wide sgRNA libraries (showing genes for which at least 2 sgRNAs changed representation in ≥ 2 folds, FDR ≤ 0.05).

Total list of candidate genes, *n* = 173	
	**First time associated with cancer platinum sensitivity/resistance, *n* = 148**
	knockout led to increase in sensitivity to platinum drugs (*n* = 109):
	*ACOX1*, *AP2M1*, *ATF6B*, *ATF7IP2*, *AUNIP*, *BPI*, *BTK*, *C14orf93*, *C17orf70*, *CAB39*, *CDC42EP3*, *CDCA5*, *CERS3*, *CERS6*, *COX7A2L*, *CRYBB1*, *DBF4*, *DDAH1*, *DDX11*, *DDX27*, *DEFA6*, *EMC2*, *EPB41L3*, *ESCO1*, *ESCO2*, *FAM160A2*, *FAM209A*, *FBXW7*, *FCHO2*, *FGFRL1*, *FOCAD*, *GFOD2*, *GJB6*, *GNAQ*, *GNGT1*, *H2BFWT*, *HERC6*, *HEYL*, *HIST1H1T*, *HMG20B*, *HMGCS2*, *HSD3B2*, *INCA1*, *KDM2B*, *KPNA2*, *KRTAP10-8*, *LEFTY1*, *LGI2*, *LMAN1*, *LPO*, *LRRC26*, *MAN2B1*, *MAP7D1*, *MAPK15*, *MARCKSL1*, *MDN1*, *MEF2A*, *METRN*, *MND1*, *MPLKIP*, *MYO1G*, *NCAPG2*, *NEUROD6*, *NGLY1*, *NIPSNAP3B*, *NPEPPS*, *NPRL2*, *ORC6*, *PAX7*, *PCGF1*, *PDIA2*, *PEF1*, *PIEZO1*, *PKD2L2*, *PLAGL2*, *PLEKHA7*, *PPP1R12A*, *PPY*, *PRF1*, *PRRC2A*, *PSMC3IP*, *PSME3*, *PTBP2*, *PVRL4*, *RAD9A*, *RASSF5*, *RGL2*, *RHNO1*, *RPA4*, *RSPH10B*, *SEMA3G*, *SMC5*, *SMOX*, *SMR3B*, *STRA13*, *SYNGR2*, *TEKT2*, *TMEM185B*, *TPM3*, *TRIM4*, *UBE2N*, *UBE2V2*, *UNC5B*, *VCY*, *VCY1B*, *WBP11*, *ZNF318*, *ZNF474*, *ZNF804A*
	knockout led to decrease in sensitivity to platinum drugs (*n* = 39):
	*AGO1*, *AGO2*, *ANKHD1*, *ATP5O*, *C14orf105*, *CAMTA2*, *CCDC102B*, *CLDN8*, *CREBL2*, *DDX26B*, *DYRK1A*, *EYA3*, *GATS*, *GDAP1L1*, *GPX8*, *LCA5L*, *LGSN*, *LOC100127983*, *MED29*, *MIS18A*, *MRPL51*, *MRPS26*, *MYOZ2*, *NDUFB10*, *NDUFC2*, *NDUFC2-KCTD14*, *NUDCD2*, *PPAPDC2*, *RBBP7*, *RECK*, *RIPPLY3*, *SLC38A2*, *SMAD2*, *TEAD2*, *TLL1*, *TMC5*, *ZBTB18*, *ZNF333*, *ZNF536*
	**Previously associated with platinum sensitivity/resistance, *n* = 25**
	knockout led to increase in sensitivity to platinum drugs (*n* = 16):
	*BRCA1*, *BRIP1*, *CDKN1B*, *ERCC4*, *EXO1*, *FANCD2*, *FANCG*, *FANCI*, *GCLM*, *LRP1*, *MAD2L2*, *MCM9*, *NBN*, *RAD51B*, *UBE2T*, *XRCC2*
	knockout led to decrease in sensitivity to platinum drugs (*n* = 9):
	*ALDH1A1*, *GAS7*, *MAPK14*, *NDUFS8*, *PMS2*, *PPP2R2A*, *STK11*, *TNFRSF12A*, *TXNRD1*

Underlined genes are involved in the regulation of MIA PaCa-2 PDAC cell sensitivity to both oxaliplatin and cisplatin.

## Data Availability

Data for this study are available from the authors upon request.

## Supplementary Materials

The following are available online: Table S1: Cell cycle and nuclear protein genes sgRNA library, Table S2: Genome-wide sgRNA library, Table S3: Analysis of sgRNA representation in CRISPR/Cas9 screening of MIA PaCa-2 pancreatic cancer cells with “cell cycle/nuclear protein genes” sgRNA library and oxaliplatin, Table S4: Analysis of sgRNA representation in CRISPR/Cas9 screening of MIA PaCa-2 pancreatic cancer cells with “genome-wide” sgRNA library and oxaliplatin, Table S5: Analysis of sgRNA representation in CRISPR/Cas9 screening of MIA PaCa-2 pancreatic cancer cells with “cell cycle/nuclear protein genes” sgRNA library and cisplatin, Table S6: GO enrichment analysis results.

## Sample Availability

Samples of the compounds are not available from the authors.

## References

[1] Sarantis P., Koustas E., Papadimitropoulou A., Papavassiliou A.G., Karamouzis M.V. (2020). Pancreatic Ductal Adenocarcinoma: Treatment Hurdles, Tumor Microenvironment and Immunotherapy. World J. Gastrointest. Oncol..

[2] Miller K.D., Siegel R.L., Lin C.C., Mariotto A.B., Kramer J.L., Rowland J.H., Stein K.D., Alteri R., Jemal A. (2016). Cancer Treatment and Survivorship Statistics, 2016. CA Cancer J. Clin..

[3] Rahib L., Smith B.D., Aizenberg R., Rosenzweig A.B., Fleshman J.M., Matrisian L.M. (2014). Projecting Cancer Incidence and Deaths to 2030: The Unexpected Burden of Thyroid, Liver, and Pancreas Cancers in the United States. Cancer Res..

[4] Ducreux M., Seufferlein T., Van Laethem J.-L., Laurent-Puig P., Smolenschi C., Malka D., Boige V., Hollebecque A., Conroy T. (2019). Systemic Treatment of Pancreatic Cancer Revisited. Semin. Oncol..

[5] Ghaneh P., Costello E., Neoptolemos J.P. (2007). Biology and Management of Pancreatic Cancer. Gut.

[6] Singh R.R., O’Reilly E.M. (2020). New Treatment Strategies for Metastatic Pancreatic Ductal Adenocarcinoma. Drugs.

[7] Singh R.R., Goldberg J., Varghese A.M., Yu K.H., Park W., O’Reilly E.M. (2019). Genomic Profiling in Pancreatic Ductal Adenocarcinoma and a Pathway towards Therapy Individualization: A Scoping Review. Cancer Treat. Rev..

[8] Kamel D., Gray C., Walia J.S., Kumar V. (2018). PARP Inhibitor Drugs in the Treatment of Breast, Ovarian, Prostate and Pancreatic Cancers: An Update of Clinical Trials. Curr. Drug. Targets.

[9] Ortíz R., Quiñonero F., García-Pinel B., Fuel M., Mesas C., Cabeza L., Melguizo C., Prados J. (2021). Nanomedicine to Overcome Multidrug Resistance Mechanisms in Colon and Pancreatic Cancer: Recent Progress. Cancers.

[10] Friedenson B. (2005). BRCA1 and BRCA2 Pathways and the Risk of Cancers Other than Breast or Ovarian. Medscape Gen. Med..

[11] Lynch H.T., Deters C.A., Snyder C.L., Lynch J.F., Villeneuve P., Silberstein J., Martin H., Narod S.A., Brand R.E. (2005). BRCA1 and Pancreatic Cancer: Pedigree Findings and Their Causal Relationships. Cancer Genet. Cytogenet..

[12] Liu Q., Liao Q., Zhao Y. (2017). Chemotherapy and Tumor Microenvironment of Pancreatic Cancer. Cancer Cell Int..

[13] Dauer P., Nomura A., Saluja A., Banerjee S. (2017). Microenvironment in Determining Chemo-Resistance in Pancreatic Cancer: Neighborhood Matters. Pancreatology.

[14] Quiñonero F., Mesas C., Doello K., Cabeza L., Perazzoli G., Jimenez-Luna C., Rama A.R., Melguizo C., Prados J. (2019). The Challenge of Drug Resistance in Pancreatic Ductal Adenocarcinoma: A Current Overview. Cancer Biol. Med..

[15] Moffat G.T., O’Reilly E.M. (2020). The Role of PARP Inhibitors in Germline BRCA-Associated Pancreatic Ductal Adenocarcinoma. Clin. Adv. Hematol. Oncol..

[16] Rebelatto T.F., Falavigna M., Pozzari M., Spada F., Cella C.A., Laffi A., Pellicori S., Fazio N. (2019). Should Platinum-Based Chemotherapy Be Preferred for Germline BReast CAncer Genes (BRCA) 1 and 2-Mutated Pancreatic Ductal Adenocarcinoma (PDAC) Patients? A Systematic Review and Meta-Analysis. Cancer Treat. Rev..

[17] Platinum Response Characteristics of Patients with Pancreatic Ductal Adenocarcinoma and a Germline BRCA1, BRCA2 or PALB2 Mutation. https://www.ncbi.nlm.nih.gov/pmc/articles/PMC7000723/.

[18] Kiyamova R., Garifulin O., Gryshkova V., Kostianets O., Shyian M., Gout I., Filonenko V. (2012). Preliminary Study of Thyroid and Colon Cancers-Associated Antigens and Their Cognate Autoantibodies as Potential Cancer Biomarkers. Biomarkers.

[19] Kostianets O., Shyian M., Sergiy D., Antoniuk S., Gout I., Filonenko V., Kiyamova R. (2012). Serological Analysis of SEREX-Defined Medullary Breast Carcinoma-Associated Antigens. Cancer Investig..

[20] Kostianets O., Shyyan M., Antoniuk S.V., Filonenko V., Kiyamova R. (2017). Panel of SEREX-Defined Antigens for Breast Cancer Autoantibodies Profile Detection. Biomarkers.

[21] CRISPR/Cas9 Technique for Identification of Genes Regulating Oxaliplatin Resistance of Pancreatic Cancer Cell Line|SpringerLink. https://link.springer.com/article/10.1007/s12668-016-0272-3.

[22] Joung J., Konermann S., Gootenberg J.S., Abudayyeh O.O., Platt R.J., Brigham M.D., Sanjana N.E., Zhang F. (2017). Genome-Scale CRISPR-Cas9 Knockout and Transcriptional Activation Screening. Nat. Protoc..

[23] Kasap C., Elemento O., Kapoor T.M. (2014). DrugTargetSeqR: A Genomics- and CRISPR-Cas9-Based Method to Analyze Drug Targets. Nat. Chem. Biol..

[24] Shalem O., Sanjana N.E., Hartenian E., Shi X., Scott D.A., Mikkelson T., Heckl D., Ebert B.L., Root D.E., Doench J.G. (2014). Genome-Scale CRISPR-Cas9 Knockout Screening in Human Cells. Science.

[25] Sarr A., Bré J., Um I.H., Chan T.H., Mullen P., Harrison D.J., Reynolds P.A. (2019). Genome-Scale CRISPR/Cas9 Screen Determines Factors Modulating Sensitivity to ProTide NUC-1031. Sci. Rep..

[26] Wang T., Wei J.J., Sabatini D.M., Lander E.S. (2014). Genetic Screens in Human Cells Using the CRISPR-Cas9 System. Science.

[27] Nurgalieva A.K., Skripova V.S., Minigulova L.F., Kiyamova R.G. (2018). Obtaining a Pancreatic Cancer Cell Line Stably Expressing Doxycycline-Dependent Endonuclease Cas9. Uchenye Zap. Kazan. Univ. Seriya Estestv. Nauk..

[28] Bruno P.M., Liu Y., Park G.Y., Murai J., Koch C.E., Eisen T.J., Pritchard J.R., Pommier Y., Lippard S.J., Hemann M.T. (2017). A Subset of Platinum-Containing Chemotherapeutic Agents Kill Cells by Inducing Ribosome Biogenesis Stress Rather than by Engaging a DNA Damage Response. Nat. Med..

[29] A Mammalian Functional-Genetic Approach to Characterizing Cancer Therapeutics|Nature Chemical Biology. https://www.nature.com/articles/nchembio.503.

[30] Riddell I.A. (2018). Cisplatin and Oxaliplatin: Our Current Understanding of Their Actions. Met. Ions. Life Sci..

[31] Poirier J.T. (2017). CRISPR Libraries and Screening. Prog. Mol. Biol. Transl. Sci..

[32] Parnas O., Jovanovic M., Eisenhaure T.M., Herbst R.H., Dixit A., Ye C.J., Przybylski D., Platt R.J., Tirosh I., Sanjana N.E. (2015). A Genome-Wide CRISPR Screen in Primary Immune Cells to Dissect Regulatory Networks. Cell.

[33] A Highly Annotated Database of Genes Associated with Platinum Resistance in Cancer|Oncogene. https://www.nature.com/articles/s41388-021-02055-2.

[34] Du J., Gu J., Li J. (2020). Mechanisms of Drug Resistance of Pancreatic Ductal Adenocarcinoma at Different Levels. Biosci. Rep..

[35] Zeng S., Pöttler M., Lan B., Grützmann R., Pilarsky C., Yang H. (2019). Chemoresistance in Pancreatic Cancer. Int. J. Mol. Sci..

[36] Constam D.B., Tobler A.R., Rensing-Ehl A., Kemler I., Hersh L.B., Fontana A. (1995). Puromycin-Sensitive Aminopeptidase: Sequence analysis, expression, and functional characterization. J. Biol. Chem..

[37] Gupta R., Somyajit K., Narita T., Maskey E., Stanlie A., Kremer M., Typas D., Lammers M., Mailand N., Nussenzweig A. (2018). DNA Repair Network Analysis Reveals Shieldin as a Key Regulator of NHEJ and PARP Inhibitor Sensitivity. Cell.

[38] Nakanishi K., Yang Y.-G., Pierce A.J., Taniguchi T., Digweed M., D’Andrea A.D., Wang Z.-Q., Jasin M. (2005). Human Fanconi Anemia Monoubiquitination Pathway Promotes Homologous DNA Repair. Proc. Natl. Acad. Sci. USA.

[39] Santidrian A.F., Matsuno-Yagi A., Ritland M., Seo B.B., LeBoeuf S.E., Gay L.J., Yagi T., Felding-Habermann B. (2013). Mitochondrial Complex I Activity and NAD^+^/NADH Balance Regulate Breast Cancer Progression. J. Clin. Investig..

[40] Wangpaichitr M., Theodoropoulos G., Nguyen D.J.M., Wu C., Spector S.A., Feun L.G., Savaraj N. (2021). Cisplatin Resistance and Redox-Metabolic Vulnerability: A Second Alteration. Int. J. Mol. Sci..

[41] Yu W., Chen Y., Dubrulle J., Stossi F., Putluri V., Sreekumar A., Putluri N., Baluya D., Lai S.Y., Sandulache V.C. (2018). Cisplatin Generates Oxidative Stress Which Is Accompanied by Rapid Shifts in Central Carbon Metabolism. Sci. Rep..

[42] ClusterProfiler: An R Package for Comparing Biological Themes Among Gene Clusters|OMICS: A Journal of Integrative Biology. https://www.liebertpub.com/doi/10.1089/omi.2011.0118.

